# Atezolizumab Plus Bevacizumab Treatment for Patients With Hepatocellular Carcinoma in the Barcelona Clinic Liver Cancer B2 Stage

**DOI:** 10.1111/jgh.70765

**Published:** 2026-09-28

**Authors:** Keishi Ouchi, Masashi Ninomiya, Mio Tsuruoka, Jun Inoue, Atsushi Hiraoka, Kosuke Sato, Kotaro Doi, Kengo Watanabe, Tomoya Sasazaki, Takayoshi Oikawa, Kei Endo, Tamami Abe, Masashi Fujita, Kazumichi Abe, Tomohiro Katsumi, Wataru Sato, Chikara Iino, Nobukazu Tanabe, Hiroshi Numao, Hiromasa Ohira, Hidekatsu Kuroda, Yoshiyuki Ueno, Atsushi Masamune

**Affiliations:** ^1^ Division of Gastroenterology Tohoku University Hospital Sendai Japan; ^2^ Institute for Excellence in Higher Education Tohoku University Sendai Japan; ^3^ Gastroenterology Center Ehime Prefectural Central Hospital Matsuyama Ehime Japan; ^4^ Division of Gastroenterology and Hepatology, Department of Internal Medicine Iwate Medical University Shiwa Iwate Japan; ^5^ Department of Gastroenterology Fukushima Medical University School of Medicine Fukushima Japan; ^6^ Department of Gastroenterology Yamagata University Faculty of Medicine Yamagata Japan; ^7^ Department of Gastroenterology, Graduate School of Medicine Akita University Akita Japan; ^8^ Department of Gastroenterology Hirosaki University Graduate School of Medicine Hirosaki Japan; ^9^ Department of Gastroenterology National Hospital Organization Sendai Medical Center Sendai Japan; ^10^ Department of Gastroenterology Aomori Prefectural Central Hospital Aomori Japan; ^11^ Tohoku Central Hospital Yamagata Japan

**Keywords:** atezolizumab plus bevacizumab, Barcelona Clinic Liver Cancer B2 stage, hepatocellular carcinoma, the efficacy of chemotherapy

## Abstract

**Background and Aim:**

The optimal treatment strategy for patients with intermediate‐stage hepatocellular carcinoma beyond the up‐to‐seven criteria (Barcelona Clinic Liver Cancer B2) remains uncertain. Although transarterial chemoembolization has long been the standard of care, its efficacy is limited in this subgroup, and systemic therapies have recently been introduced. We aimed to explore treatment modalities associated with survival beyond that predicted by a previously validated prognostic model.

**Methods:**

We retrospectively analyzed 113 patients diagnosed with BCLC‐B2 stage hepatocellular carcinoma between 2017 and 2024. Predicted survival was calculated using a previously established prognostic model, and treatment benefits were evaluated by comparing observed survival with predicted outcomes. Logistic and linear regression analyses were performed to identify factors associated with survival gain.

**Results:**

The median observed survival was 26.0 months, compared with a model‐predicted survival of 24.7 months. Treatment with atezolizumab plus bevacizumab was significantly associated with survival exceeding model predictions (odds ratio: 3.77, *p* = 0.0065). Linear regression analysis confirmed that atezolizumab plus bevacizumab was independently associated with a greater survival benefit (β = 0.192, *p* = 0.0084). Subgroup analysis demonstrated that this association was particularly pronounced in patients with high tumor burden (tumor number plus size ≥ 11), whereas no significant associations were observed for lenvatinib, STRIDE, or cabozantinib.

**Conclusions:**

Atezolizumab plus bevacizumab was associated with survival beyond that predicted by a prognostic model in patients with BCLC‐B2 stage hepatocellular carcinoma, particularly those with high tumor burden. These findings should be considered exploratory and associative rather than evidence of a causal treatment effect.

**Abbreviations**
ALBI: albumin‐bilirubin
ATZ + Bev: atezolizumab plus bevacizumab
BCLC: Barcelona Clinic Liver Cancer
CABO: cabozantinib
HCC: hepatocellular carcinoma
IQR: interquartile range
LEN: lenvatinib
mALBI: modified ALBI
OR: odds ratio
STRIDE: durvalumab plus tremelimumab regimen (single tremelimumab regular interval durvalumab)
TACE: transarterial chemoembolization

## 1 Introduction

Currently, the management of hepatocellular carcinoma (HCC) is guided by recommendations from the American Association for the Study of Liver Diseases and European Association for the Study of the Liver. In these guidelines, HCC is staged according to the Barcelona Clinic Liver Cancer (BCLC) system [1, 2]. The BCLC staging system classifies HCC into five categories, with the intermediate stage (BCLC B) defined as multinodular disease without vascular invasion or extrahepatic spread [3, 4].

Between 2000 and the early 2010s, transarterial chemoembolization (TACE) was established as the standard therapeutic approach for patients with BCLC B stage disease [5, 6]. Although TACE has long been regarded as the standard of care, its therapeutic outcomes are heterogeneous because of variations in tumor burden, underlying liver function, operator expertise, and institutional infrastructure. Moreover, repeated TACE sessions can lead to the deterioration of liver function, posing a significant clinical limitation [7, 8, 9]. More recently, the concept of TACE unsuitability has been introduced, acknowledging that even in intermediate‐stage HCC, TACE may not be effective depending on tumor size, morphology, or liver function [10, 11].

To address this heterogeneity, Bolondi's subclassification of BCLC stage B was subsequently refined by Kudo et al., who proposed the Kinki criteria. In this framework, B1 is defined as Child–Pugh A–B7 with tumors within the up‐to‐seven criteria, B2 as Child–Pugh A–B7 with tumors beyond the up‐to‐seven criteria, and B3 as Child–Pugh B8–9 irrespective of tumor burden. Because B2 stage disease can be considered TACE‐unsuitable, systemic therapy—as well as strategies combining systemic and locoregional therapies aimed at conversion—are now recommended [12, 13, 14, 15].

The TACTICS trial, reported in 2020, compared TACE alone with TACE plus sorafenib in patients with unresectable HCC and demonstrated that the combination therapy significantly prolonged disease control in patients with BCLC B stage disease. This finding challenged the long‐standing paradigm of TACE monotherapy and paved the way for TACE–systemic therapy combinations [16].

In 2018, lenvatinib (LEN) became available, followed in 2020 by atezolizumab plus bevacizumab (ATZ + Bev), the first regimen incorporating an immune checkpoint inhibitor for HCC [17, 18]. Subsequently, several immune checkpoint inhibitor‐based regimens have been approved. However, it remains unclear whether these agents improve survival, particularly in patients with BCLC B2 stage disease. Furthermore, in real‐world practice, treatment rarely consists of a single modality; instead, multiple systemic and locoregional therapies are often combined or used sequentially depending on disease status, complicating the evaluation of individual treatment effectiveness.

Given this background, we developed a prognostic model to estimate survival in patients diagnosed with BCLC B stage disease before 2017 [19]. Whether immunotherapy‐based systemic therapies can overcome the historically poor prognosis predicted by TACE‐era models remains unclear. Therefore, using this prognostic model as a reference, we aimed to evaluate which treatment modalities contribute to survival beyond predicted outcomes and to identify therapies associated with prolonged survival in HCC patients with B2 stage.

## 2 Methods

### 2.1 Study Design and Participants

In this retrospective cohort study, a total of 113 patients with HCC were enrolled between 2017 and 2024 at five liver centers in Japan (Hirosaki University Hospital, Akita University Hospital, Yamagata University Hospital, Tohoku University Hospital, and Ehime Prefectural Central Hospital). The HCC stage was evaluated according to the BCLC classification, and all patients diagnosed with B2 stage disease were included. They survived for at least 3 months after initiation of treatment following the diagnosis of BCLC B2 stage. The index date was defined as the date of treatment initiation after BCLC B2 diagnosis. To reduce immortal bias, a landmark analysis was additionally performed. The landmark time point was set at 3 months after the index date, and only patients who were alive at the 3‐month landmark were included in this study. Overall survival was defined as the duration between the index date (treatment initiation after BCLC B2 stage diagnosis) and death. The clinical information of the patients was obtained from their medical records, and they were followed up to determine their vital status using medical information sheets from the respective hospitals. All therapies were permitted; however, none of the patients underwent liver transplantation.

This study was approved by the Institutional Review Board of Tohoku University Hospital (2024‐1‐999) and was conducted according to the principles of the Declaration of Helsinki (Fortaleza Revision, 2013).

### 2.2 Data Collection

HCC nodules were diagnosed using contrast‐enhanced computed tomography or magnetic resonance imaging, and the following parameters were recorded: number of tumor nodules, diameter of the largest nodule, and presence of vascular invasion. The following clinical and biochemical parameters were collected: age, gender, etiology, Eastern Cooperative Oncology Group performance status, total bilirubin, aspartate aminotransferase, alanine aminotransferase, albumin, platelet count, prothrombin time, α‐fetoprotein, des‐γ‐carboxyprothrombin, tumor size, number of nodules, albumin‐bilirubin (ALBI) score, modified ALBI grade, Child–Pugh score, and prior treatment. The ALBI score was calculated using the formula: linear predictor = [log_10_(total bilirubin [mg/dL]x17.1) × 0.66] + (albumin [g/dL]x10 × −0.085). The cut points for the modified ALBI grade were as follows: x ≤ −2.60 (Grade 1); −2.60 < x < −2.27 (Grade 2a); −2.27 ≤ x ≤ −1.39 (Grade 2b); and x > −1.39 (Grade 3) [20, 21]. Newly approved systemic therapies were categorized into five treatments: ATZ + Bev; LEN; durvalumab plus tremelimumab (STRIDE); cabozantinib (CABO); other chemotherapies (sorafenib, regorafenib, ramucirumab, and/or investigational agents). For each treatment, treatment exposure was defined based on whether the patient received the therapy at any time after the diagnosis of BCLCB2 stage. Accordingly, these variables were treated as postdiagnosis treatment experiences (yes/no) occurring after the index date. Continuous variables are presented as medians (interquartile ranges [IQRs]), and categorical variables are presented as counts (numbers). No missing data were present for the variables included in the analyses.

### 2.3 Statistical Analysis

Patient survival probability was analyzed using the Kaplan–Meier method. Patients alive at the end of follow‐up were censored at the date of the last follow‐up. Survival prediction was performed using the model reported by Ninomiya et al. The 50% survival duration (months) was expressed as follows: EXP [4.02580 + (−0.0086253 × age) + (−0.34667 × recurrence status [0 = naïve/1 = recurrence]) + (−0.034962 × number of nodules) + (−0.079447 × largest nodule size [cm]) + (−0.21696 × total bilirubin [mg/dL]) + (0.27912 × albumin [g/dL]) + (−0.00014741 × α‐fetoprotein [ng/dL])] × (−log_0.5_)^0.67250^ [19].

Observed survival time was compared with predicted survival time using a paired *t*‐test because both values represented paired measurements from the same patient. A logistic regression model was constructed, with the outcome variable defined as whether the observed survival exceeded the predicted survival (coded as one when observed survival was longer). Treatment modalities were used as explanatory variables, specifically whether the patients had received ATZ + Bev, LEN, STRIDE, CABO, or other chemotherapy. Variables retained in the final model were subjected to multivariate logistic regression analysis.

In addition to the logistic regression analysis, we examined the magnitude of benefit by calculating the ratio of observed‐to‐predicted survival time. This was expressed as log (observed survival time/predicted survival time), which was then used as the dependent variable in a multiple linear regression analysis to assess factors associated with prolonged survival. To identify explanatory variables associated with survival length, we first performed variable selection using least absolute shrinkage and selection operator (LASSO) regression. Candidate variables included clinical and treatment‐related factors recorded after the diagnosis of BCLCB2 stage. Variables selected by LASSO were then entered into a multiple linear regression model, with log (observed survival time/predicted survival time) as the dependent variable, to determine independent factors associated with greater‐than‐expected survival benefit. Treatment modalities were used as explanatory variables for the 87 patients whose survival times were confirmed.

Logistic regression analysis was performed using JMP Student Edition 18.2.0 (SAS Institute, Cary, NC, USA). LASSO regression and multiple linear regression analysis were performed using RStudio (RStudio, Boston, MA, USA). Statistical significance was defined as a two‐sided *p*‐value < 0.05.

## 3 Results

### 3.1 Characteristics of the Patients in This Cohort Study

A total of 113 patients with BCLC B2 stage disease were included in this study (Table 1). The median age was 73 years (IQR, 67–78), and the majority of the patients were men (88/113, 77.8%). The most common etiology was nonviral infection (*n* = 66), followed by hepatitis C virus infection (*n* = 37). Most patients maintained a good performance status, with an Eastern Cooperative Oncology Group performance status of zero in 113 cases (100%). The median serum albumin level was 3.7 g/dL (IQR, 3.4–4.0), and the median total bilirubin concentration was 0.9 mg/dL (IQR, 0.7–1.3). The median ALBI score was −2.43 (IQR, −2.69 to −2.06), corresponding to a modified ALBI grade of 1 in 39 patients, 2a in 30, 2b in 42, and 3 in two. The Child–Pugh scores were 5, 6, and 7 in 53, 41, and 19 patients, respectively.

**TABLE 1:** Description of the patients (*n* = 113).

Patient characteristics	B2 stage (*n* = 113)
Age, years	73 (67–78)
Gender, male/female	88/25
Etiology, HCV/HBV/HCV + HBV/nonBnonC	37/9/1/66
ECOG, PS 0/1	113/0
Naïve/recurrence	59/54
Total bilirubin, mg/dL	0.9 (0.7–1.3)
AST, U/L	41 (26–61)
ALT, U/L	34 (20–48.5)
Albumin, g/dL	3.7 (3.4–4.0)
Platelets, ×10^4^/μL	14.1 (10.3–18.6)
Prothrombin time, %	95.0 (85.25–103.7)
AFP, ng/dL	13.8 (5.0–298.65)
DCP, mAU/mL	317 (39.25–2740.5)
ALBI score	−2.43 (−2.69–−2.06)
mALBI grade, 1/2a/2b/3	39/30/42/2
Child–Pugh score, 5/6/7	53/41/19
Size of the largest nodules, cm	3.8 (2.0–6.5)
< 1/1–2/2–3/3–4/4–6/6–10/≥ 10	0/23/19/15/21/21/14
Number of nodules	6 (4–7.5)
2/3/4/5/6–7/8–9/≥ 10	11/15/8/15/36/1/27
The average number of treatments after B2 stage	4.0
Treatment exposure after B2 stage	
Atezolizumab + bevacizumab	42
Lenvatinib	42
Durvalumab + tremelimumab	12
Cabozantinib	11
Other chemotherapies	31
Transarterial (chemo)embolization	86
Local ablation	22

Abbreviations: AFP, α‐fetoprotein; ALBI, albumin‐bilirubin; ALT, alanine aminotransferase; AST, aspartate aminotransferase; DCP, des‐γ‐carboxyprothrombin; ECOG, Eastern Cooperative Oncology Group; HBV, hepatitis B virus; HCV, hepatitis C virus; mALBI, modified ALBI; PS, performance status.

The median diameter of the largest tumor was 3.8 cm (IQR, 2.0–6.5). Regarding the distribution of tumor size, 23 patients had tumors measuring 1–2 cm, 19 had tumors 2–3 cm, 15 had tumors 3–4 cm, 21 had tumors 4–6 cm, 21 had tumors 6–10 cm, and 14 had tumors ≥ 10 cm. The median number of nodules was six (IQR, 4–7.5), with 27 patients presenting ≥ 10 nodules.

Regarding treatments exposure after B2 stage, the average number showed 4.0 and the systemic therapies included ATZ + Bev (*n* = 42), LEN (*n* = 42), STRIDE (*n* = 12), and CABO (*n* = 11). Additionally, 31 patients received other chemotherapies.

Figure 1a shows the Kaplan–Meier curve for the entire cohort and stratified by treatment era (2017–2020 and 2021–2024). The median overall survival was 26.0 months (95% confidence interval [CI], 21.3–35.0), with estimated 1‐, 2‐, and 3‐year survival rates of 77.0%, 56.1%, and 39.2%, respectively. Survival curves for the two treatment eras are also presented to illustrate potential temporal differences in overall survival during the study period. There was no significant difference in overall survival between the two treatment eras (log‐rank *p* = 0.2208).

**FIGURE 1:**
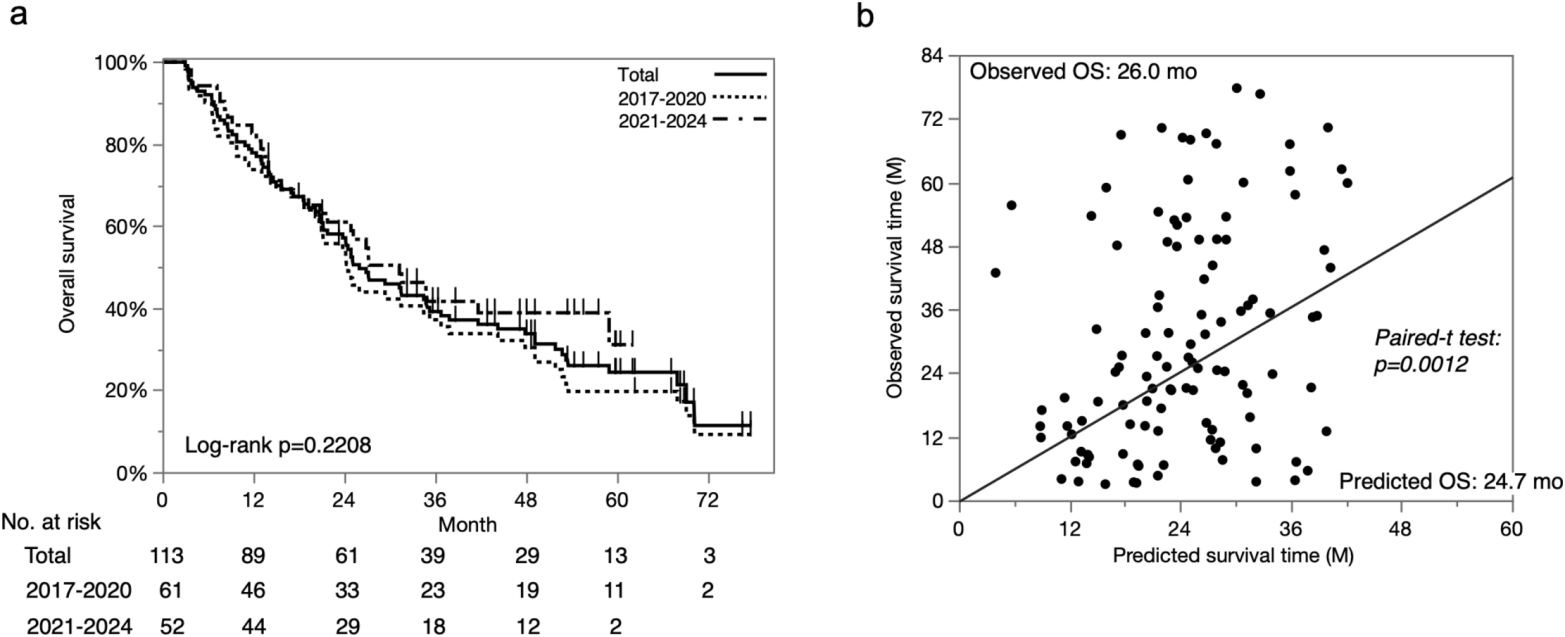
(a) Kaplan–Meier curves for overall survival in patients with hepatocellular carcinoma at Barcelona Clinic Liver Cancer B2 stage. (b) Comparison between the predicted survival times derived from the prognostic model and observed survival times.

Figure 1b shows the comparison between the predicted survival times derived from the prognostic model and observed survival times. The scatterplot demonstrates the relation between the model predictions and actual outcomes. The median predicted survival time according to the prognostic model was 24.7 months, whereas the median observed overall survival was 26.0 months (paired *t*‐test, *p* = 0.0012).

### 3.2 Effect of Treatment Modalities on Survival Beyond the Predicted Outcome

We compared the predicted survival duration with observed survival duration to identify treatment modalities associated with prolonged survival. In the logistic regression analysis, treatment with ATZ + Bev was significantly associated with prolonged survival beyond that predicted by the model (odds ratio [OR], 3.77; 95% CI, 1.43–10.91; *p* = 0.0065). In contrast, treatment with LEN, STRIDE, CABO, or other chemotherapy regimens was not significantly associated with observed survival exceeding predicted survival (Table 2). Exposure to ATZ + Bev was independently associated with a significantly higher likelihood of achieving longer‐than‐predicted survival and a median observed survival duration exceeding the predicted survival, as illustrated in the scatterplot (observed OS; 37.9 months vs. predicted; 25.3 months, paired *t*‐test, *p* < 0.0001) (Figure 2), whereas no significant associations were indicated for other treatment modalities. These findings suggest that experience with treatment using ATZ + Bev was associated with a greater‐than‐expected survival benefit among patients with B2 stage. Baseline clinical characteristics according to ATZ + Bev exposure are presented in Table S1. No significant differences were observed between ATZ + Bevexposed and unexposed groups except for DCP level.

**TABLE 2:** Logistic regression analysis of treatment modalities associated with survival benefit.

Variable	Odds ratio	95% CI	*p*
Atezolizumab + bevacizumab	3.773	1.44–10.91	0.0065
Lenvatinib	1.504	0.62–3.72	0.3698
Durvalumab + tremelimumab	2.035	0.37–15.78	0.4217
Cabozantinib	1.957	0.25–41.39	0.5528
Other chemotherapy	1.283	0.50–3.32	0.6012

**FIGURE 2:**
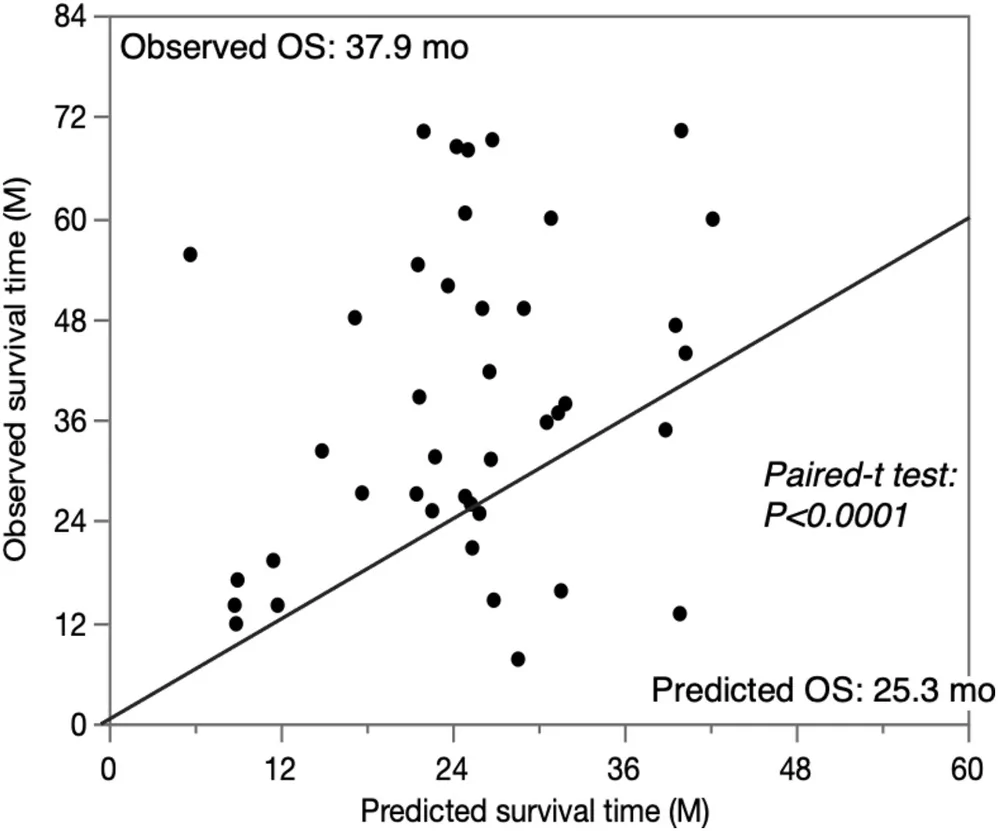
Scatterplot comparing observed survival for individual patients and predicted survival derived from the prognostic model with the exposure of ATZ + Bev. The diagonal dashed line indicates equality between observed and predicted survival.

We further stratified the cohort by tumor burden (sum of tumor number and maximum diameter), classifying patients into those with scores of 7–11 and those with scores ≥ 11. The logistic regression analyses were conducted separately for each subgroup using the same explanatory variables as in the overall analysis. The upper panel of Table 3 summarizes the results for patients with tumor burdens of 7–11, whereas the lower panel presents the results for those with tumor burdens ≥ 11. In patients with a tumor burden of 7–11, no treatments were significantly associated with prolonged survival. But, in patients with a tumor burden ≥ 11, just ATZ + Bev was the only treatment modality significantly associated with a survival benefit (Table 3).

**TABLE 3:** Logistic regression analysis of treatment modalities associated with survival benefit according to tumor burden (tumor number plus size 7–11 in upper and ≥ 11 in lower).

Variable	Odds ratio	95% CI	*p*
Atezolizumab + bevacizumab	2.03	0.619–7.110	0.2447
	15.31	2.279–311.9	0.0031
Lenvatinib	1.30	0.401–4.322	0.6612
	1.64	0.354–7.505	0.5211
Durvalumab + tremelimumab	2.64	0.308–56.73	0.3951
	2.10	0.114–59.91	0.6093
Cabozantinib			
	0.29	0.00820–10.53	0.4631
Other chemotherapy	0.83	0.241–2.783	0.7565
	2.25	0.472–11.99	0.3106

### 3.3 Extent of Survival Benefit According to Treatment Modality

To further evaluate the magnitude of survival benefit, we analyzed the log‐transformed ratio observed‐to‐predicted survival duration using multiple linear regression analysis in 87 patients with confirmed survival outcomes. Variable selection was performed using LASSO regression, and selected variables were subsequently included in the multivariable linear regression model.

As shown in Table 4, experience with ATZ + Bev was significantly associated with a higher log (observed survival time/predicted survival time) (coefficient [β], 0.192; 95% CI, 0.075–0.34; *p* = 0.0084), indicating a greater‐than‐expected survival benefit. In contrast, treatment with LEN, STRIDE, or other chemotherapy regimens was not significantly associated with the survival benefit. CABO was not retained in the final LASSO model, likely reflecting its limited independent contribution to prediction, which may in part be attributable to the relatively small number of treated patients and overlap with other treatment variables.

**TABLE 4:** Results of multiple linear regression analysis for the log‐transformed ratio of actual to predicted survival according to treatment modalities.

Prior treatment	Coefficient (β)	95% CI	*p*
Atezolizumab + bevacizumab	0.192	0.075–0.34	0.0084
Lenvatinib	0.0331	−0.020–0.28	0.4640
Durvalumab + tremelimumab	0.0733	−0.059–0.38	0.3272
Cabozantinib			
Other chemotherapy	0.0740	−0.0062–0.32	0.1779
Model performance			
*R* ^2^/Adjusted *R* ^2^	0.121/0.0890		

Subgroup analysis stratified by tumor burden, based on the sum of tumor number and size (7–11 vs. ≥ 11), is presented in Table 5. In patients with higher tumor burden (tumor number plus size ≥ 11), treatment with ATZ + Bev remained significantly associated with increased log (observed survival time/predicted survival time) (β, 0.262; 95% CI, 0.11–0.52; *p* = 0.0011). However, no significant association was observed in patients with lower tumor burden (tumor number plus size 7–11). Other treatment modalities were not significantly associated with survival benefit in either subgroup.

**TABLE 5:** Results of multiple linear regression analysis for the log‐transformed ratio of actual to predicted survival according to treatment modalities (tumor number plus size 7–11 in upper and ≥ 11 in lower).

Prior treatment	Coefficient (β)	95% CI	*p*
Atezolizumab + bevacizumab	0.0463	−0.030–0.31	0.4130
	0.262	0.11–0.52	0.0011
Lenvatinib			
Durvalumab + tremelimumab	0.115	−0.080–0.46	0.2317
Cabozantinib			
Other chemotherapy	0.0158	−0.17–0.45	0.6485
	0.0238	−0.030–0.44	0.1517
Model performance			
*R* ^2^/Adjusted *R* ^2^	0.0752/0.0312		
	0.231/0.195		

## 4 Discussion

In this study, we applied a validated prognostic model to patients with BCLC B2 stage disease to evaluate the impact of different treatment modalities on survival beyond the predicted prognosis. The predicted median survival of 24.7 months was comparable to the observed median survival of 26.0 months, suggesting that the model provides an appropriate benchmark for assessing treatment benefits in this cohort.

Experience with ATZ + Bev was consistently associated with a greater‐than‐expected survival benefit in HCC patients with B2 stage, as demonstrated by both logistic regression and linear regression analyses using the log‐transformed ratio of observed‐to‐predicted survival. Importantly, the survival prediction model used in this analysis was developed based on patient cohorts treated predominantly in the era when TACE constituted the mainstay of therapy for intermediate‐stage disease.

Under TACE‐centered treatment strategies, patients with high tumor burden typically experience limited survival benefit due to reduced treatment efficacy, frequent repetition of procedures, and progressive deterioration of liver function. As a result, predicted survival for patients with extensive tumor burden is inherently poor in such models. Against this historical background, the observation that ATZ + Bev was associated with survival substantially exceeding these predictions—particularly in patients with high tumor burden—suggests an association with survival beyond that predicted by the historical TACE‐era model.

The combination of immune checkpoint inhibition and vascular endothelial growth factor blockade may confer a distinct advantage in patients with extensive disease, for whom locoregional therapies alone are often insufficient. Bev‐induced vascular normalization may enhance immune cell trafficking and improve drug delivery within large or multifocal tumors, whereas ATZ may restore antitumor immune surveillance in an immunosuppressive tumor microenvironment. These effects are unlikely to be captured by prognostic models derived from TACE‐era cohorts and may explain why the survival benefit associated with ATZ + Bev was most evident in patients with higher tumor burden. While the absence of a significant association with LEN, STRIDE, or CABO may reflect differences in mechanisms of action, patient selection, treatment timing, and limited statistical power. In particular, the relatively small number of patients receiving STRIDE and CABO may have reduced the ability to detect significant associations. Further studies with larger cohorts are warranted to clarify the survival impact of these therapies.

Collectively, these findings suggest that immunotherapy‐based combinations may redefine survival expectations for selected patients with B2 stage disease, particularly those with tumor characteristics historically associated with poor outcomes under TACE‐dominant treatment paradigms.

To quantify the magnitude of survival benefit beyond conventional survival comparisons, we used the log‐transformed ratio of observed‐to‐predicted survival time as a continuous outcome variable. This approach allows evaluation of how much longer patients survived relative to expectations derived from prognostic models, rather than merely assessing survival probabilities at fixed time points. A positive coefficient in this model indicates that observed survival exceeded predicted survival. For example, the regression coefficient of 0.192 associated with ATZ + Bev corresponds to approximately a 21% increase in observed survival relative to predicted survival (exp[0.192] = 1.21). Similarly, in patients with high tumor burden of up‐to‐eleven out, the coefficient of 0.262 suggests an even greater relative survival extension. These findings provide a clinically interpretable measure of treatment benefit that complements traditional hazard‐based or binary outcome analyses. Importantly, the concordant results across logistic regression (binary outcome) and linear regression (continuous outcome) analyses strengthen the robustness of the observed association and reduce the likelihood that the findings are driven by model‐specific artifacts.

Several limitations of this study should be acknowledged. First, this was an observational analysis, and treatment allocation was not randomized. Although landmark analysis and multivariable modeling were used to mitigate immortal time bias and confounding, residual immortal time bias may remain because patients initiating systemic therapies later during follow‐up were classified according to ever exposure after BCLCB2 diagnosis. A time‐dependent Cox regression would provide a more rigorous evaluation and should be considered in future studies. Second, treatment exposure was defined as receipt of each chemotherapy after BCLCB2 diagnosis, which inherently reflects postbaseline events. Although this reflects real‐world clinical practice, treatment crossover and sequencing effects may have influenced survival outcomes. Finally, the predicted survival model used as a reference may not fully capture all prognostic factors influencing individual patient outcomes. Consequently, the observed‐to‐predicted survival ratio should be interpreted as a relative, rather than absolute, measure of treatment benefit. Furthermore, because the prognostic model was developed from a historical cohort treated predominantly during the TACE era, improvements in the overall management of HCC over time may have contributed to the observed survival gains. Therefore, the observed‐to‐predicted survival comparison should be interpreted as an exploratory measure of association rather than definitive evidence of treatment efficacy.

In conclusion, ATZ + Bev was associated with survival beyond that predicted by the validated prognostic model, particularly in patients with high tumor burdens, suggesting that treatment selection for BCLC B2 stage disease should consider both tumor characteristics and the potential for survival gain beyond predicted outcomes.

## Funding

The authors have nothing to report.

## Ethics Statement

This study adhered to the principles of the Declaration of Helsinki (Fortaleza Revision, 2013). This study was reviewed and approved by the Institutional Review Board of Tohoku University Hospital (Approval Number: 2024‐1‐999).

## Consent

Because of the retrospective, observational nature of the study, the Institutional Review Board of Tohoku University Hospital waived the requirement for written informed consent. The identifying data of enrolled patients were deleted, and the authors did not have access to any individual‐level data.

## Conflicts of Interest

The authors declare no conflicts of interest.

## Supporting information


**Table S1:** Description of the patients with ATZ + Bev exposed and unexposed.

## Data Availability

All data generated or analyzed during this study are included in this article. Further inquiries can be directed to the corresponding authors.
