# Supplementary material for: Atezolizumab Plus Bevacizumab Treatment for Patients With Hepatocellular Carcinoma in the Barcelona Clinic Liver Cancer B2 Stage
Source: J Gastroenterol Hepatol. 2026 Sep 28;41(11):3567–74. doi: 10.1111/jgh.70765 (PMC13731034; doi:10.1111/jgh.70765)
Supplement: Supplementary file 1 [file JGH-41-3567-s001.docx]

**Supplement Table1.** Description of the patients with ATZ+Bev exposed and unexposed.

| Patient Characteristics | Exposed (n=42) | Unexposed (n=71) | p-value |
| --- | --- | --- | --- |
| Age, years | 71 (66 -77) | 73 (67-78) | 0.400 |
| Gender, Male/Female | 32/10 | 56/15 | 0.741 |
| Etiology, HCV/HBV/HCV+HBV/nonBnonC | 11/4/0/27 | 26/5/1/39 | 0.331 |
| Total bilirubin, mg/dL | 0.85 (0.6 -1.3) | 0.9 (0.7-1.3) | 0.912 |
| AST, U/L | 39.5 (23.5 – 58.75) | 42 (28 – 61) | 0.626 |
| ALT, U/L | 32 (17 – 47.5) | 36 (21 – 54) | 0.410 |
| Albumin, g/dL | 3.85 (3.5-4.0) | 3.7 (3.3-4.1) | 0.299 |
| Platelets, x10^4^/μL | 14.1 (10.1-17.9) | 13.9 (10.5-19.1) | 0.979 |
| Prothrombin time, % | 97.0 (84.7-110.9) | 94.3 (85.6-101.6) | 0.407 |
| AFP, ng/dL | 8.7 (5.1-31.7) | 23.8 (4.4-469) | 0.0677 |
| DCP, mAU/mL | 112.5 (36.75-828.5) | 756.5 (72.25-4993) | 0.0148 |
| ALBI score | -2.48 (-2.70 - -2.13) | -2.35 (-2.68- -1.96) | 0.351 |
| mALBI grade, 1/2a/2b/3 | 14/15/13/0 | 25/15/29/2 | 0.259 |
| Child-Pugh score, 5/6/7 | 21/14/7 | 32/27/12 | 0.862 |
| Size of the largest nodules, cm | 3.75 (1.97-5.6) | 4 (2.1-7) | 0.314 |
| <1/1-2/2-3/3-4/4-6/6-10/≥10 | 0/10/7/5/10/6/4 | 0/13/12/10/11/15/10 |  |
| Number of nodules | 6 (4-10) | 6 (4-7) | 0.336 |
| 2/3/4/5/6-7/8-9/≥10 | 2/7/4/3/13/1/12 | 9/8/4/12/23/0/15 |  |
| 7-11 criteria | 24/18 | 43/28 | 0.721 |

AST, aspartate aminotransferase; ALT, alanine aminotransferase; AFP, α-fetoprotein; DCP, des-γ-carboxyprothrombin; ALBI, albumin-bilirubin; mALBI, modified ALBI; HBV, hepatitis B virus; HCV, hepatitis C virus
